# Habits of Genital Hygiene and Sexual Activity among Women with Bacterial Vaginosis and/or Vulvovaginal Candidiasis

**DOI:** 10.1055/s-0041-1741536

**Published:** 2022-02-25

**Authors:** Marcela Grigol Bardin, Paulo César Giraldo, Cristina Laguna Benetti-Pinto, José Marcos Sanches, Camila Carvalho de Araujo, Rose Luce Gomes do Amaral

**Affiliations:** 1 Department of Gynecology and Obstetrics, Universidade Estadual de Campinas, Campinas, SP, Brazil; 2 Division of Physiotherapy, Universidade Estadual de Campinas, Campinas, SP, Brazil; 3 Department of Morphology and Genetics, Universidade Federal de São Paulo, São Paulo, SP, Brazil

**Keywords:** bacterial vaginosis, candida albicans, hygiene, sexual behavior, disbiosis, vaginose bacteriana, candida albicans, higiene, comportamento sexual, disbiose

## Abstract

**Objective** To evaluate genital hygiene among women with and without bacterial vaginosis (BV) and/or vulvovaginal candidiasis (VVC).

**Methods** A cross-sectional study of reproductive-aged women who underwent gynecological and laboratory tests and fulfilled a genital hygiene questionnaire.

**Results** This study evaluated 166 healthy controls and 141 women diagnosed with either BV (n = 72), VVC (n = 61), or both (n = 8). The use of intimate soap and moist wipes after urination was more frequent among healthy women ( *p* = 0.042 and 0.032, respectively). Compared to controls, bactericidal soap was more used by women with BV ( *p* = 0.05).

**Conclusion** Some hygiene habits were associated to BV and/or VVC. Clinical trials should address this important issue in women's health.

## Resumo

**Objetivo** Avaliar a higiene genital de mulheres com e sem vaginose bacteriana (VB) e/ou candidíase vulvovaginal (CVV).

**Métodos** Estudo transversal com mulheres em idade reprodutiva submetidas a exames ginecológicos e laboratoriais e preenchimento de questionário de higiene genital.

**Resultados** Este estudo avaliou 166 controles saudáveis e 141 mulheres com diagnóstico de VB (n = 72), VVC (n = 61) ou ambas (n = 8). O uso de sabonete íntimo e lenços umedecidos após a micção foram hábitos mais frequentes entre mulheres saudáveis ( *p* = 0,042 e 0,032, respectivamente). Em comparação com os controles, o sabonete bactericida foi mais usado por mulheres com VB ( *p* = 0,05).

**Conclusão** Alguns hábitos de higiene foram associados à VB e/ou VVC. Os ensaios clínicos devem abordar esta questão importante na saúde da mulher.

## Introduction

Vulvovaginal candidiasis (VVC) and bacterial vaginosis (BV) are among the most prevalent conditions seen by medical doctors assisting women with several vulvovaginal symptoms. The prevalence of VVC and BV may vary in function of endogenous and exogenous factors, leading to the development of one or both of these conditions in 60% of women in reproductive age. 1 2 However, many of the women suffering with vulvovaginal disorders (VDs) do not match the well-known risk factors. 3

Bacterial vaginosis, which is the most frequently cited cause of vaginal discharge and malodor, is associated with an increased risk of sexually transmitted disease (human papillomavirus [HPV], human immunodeficiency virus [HIV], pelvic inflammatory disease [PID]) and a number of other adverse reproductive outcomes. 4 An increased vaginal pH and the replacement of vaginal lactobacilli by *Gardnerella vaginalis* and anaerobic gram-negative rods characterizes this VD. 5 Vulvovaginal candidiasis is an extremely common infection in women of childbearing age of all strata of society, the second most common cause of vaginitis in the United States and the most common cause in Europe, and it has a high negative impact over women's comfort and well-being. 6

Some genital hygiene behaviors and/or sexual practices might represent potential mechanisms for facilitating the installation of one of these conditions. Certain vulvar cleansing agents and vaginal douching may affect the vulvovaginal ecology through alteration of pH or bactericidal effects on the normal lactobacilli and, so, predispose to BV. 7 Feminine hygiene products (such as women's blades, sprays, showers, yeast creams, and pubic hair removal oils) are extensively used worldwide, even though they could modify the genital environment; however, unfortunately, this is still poorly studied. Even if feminine hygiene products are not causative of VD, the use of these products could cause symptoms that mimic VD, such as discharge or irritation, or may mask symptoms of vulvar and vaginal infections, misleading the diagnosis and treatment.

The literature suggests that receptive oral sex could introduce abnormal flora or lactobacilli phages into the vagina, or that a salivary mediator could cause alteration in the vaginal flora and favor VVC installation. 8 9 Therefore, the goal of the present study was to describe the genital hygiene and sexual habits among reproductive-aged women and to look for possible associations between the diagnosis of VD and those practices.

## Methods

This is a cross-sectional study of reproductive-aged women attended at Hospital da Mulher Professor Doctor José Aristodemo Pinotti-CAISM-UNICAMP. The period of data collection was between February, 2013 and May, 2014, after approval of the institutional ethics committee (CAAE: 04945812.5.0000.5404). In the first step of patient selection, the main researcher checked on charts of patients who were in the waiting room. The inclusion (to be in reproductive age [considered from 18–45 years old] and to have preserved ovaries function) and exclusion criteria (diagnose of sexual transmitted diseases, genital hygiene orientation received previously in our services, previous gynecological cancer, diagnose of diabetes or other immunosuppressive disease, cognitive difficulties, antibiotic or vaginal medication use in the 15 days prior to selection) were accessed in order to appoint eligible participants. In total, 360 patients were selected and invited to participate in the study. One hundred and ninety cases were elected at the family planning and 170 cases at the genital infection outpatient clinics. All women who fulfilled the inclusion criteria and signed the informed consent form were enrolled in the study. They answered questions about sociodemographic and gynecological characteristics and fulfilled a self-reported standardized questionnaire containing habits of genital hygiene, sexual activity, and related care. They were guided to a gynecological examining room where signals of vaginal disorders were searched, and vaginal sampling for clinical and microbiological diagnosis of BV and/or VVC were collected. Infections such as HIV, hepatitis B and C, and syphilis were excluded by serology, HPV by oncologic colpocytology and *Neisseria gonorrhoeae* by culture in Thayer-Martin medium. In case of clinical suspicion of infection by *Chlamydia trachomatis,* patients were excluded. There were no cases of clinical genital herpes infection. In order to compound the healthy group, all cases presenting vaginal microflora missing *Lactobacilli* or presenting more than 10 leucocytes per immersion oil field (x 1,000) or with severe cytolysis in the microscopy were excluded.

## Questionnaire

Because there is no validated questionnaire for genital hygiene and daily care available in the scientific literature so far, some researchers in this field developed specific questions in order to understand these important habits among women. However, the questionnaires used in the consulted literature 7 9 10 11 12 not only are not validated, but also lack valuable information such as frequency of genital washing, technique used to have pubic hair removed, among others that might be relevant to this research. The developed tool's structure is divided into the following 6 main domains: 1. Genital cleansing and washing; 2. Sexual activity and related care; 3. Genital hair removal aspects; 4. Tattooing and piercing; 5. Pad, tampon, and other products used during menstruation, and 6. Type and fabric of most used clothing and underwear. The domains add up to 60 questions that can be answered categorically (eg: yes or no, never, sometimes, frequently or always, or by checking directly the product used on the query care). Before beginning data collection, the tool was tested in a pilot study and adjusted as necessity was pointed out either by patients or professionals who analyzed the questionnaire' answers. In addition, Cronbach's alpha coefficients were calculated to assess the internal consistency of the questionnaire. Only the questions scored with an almost perfect agreement level (> 0.80) were used in the analysis of the present study, in order to assure an adequate test-retest reliability to assess the genital hygiene and sexual behavior of women with and without vulvovaginal diagnosis. This study analyzed questions of the domains 1, 2, and 3 (genital cleansing and washing, sexual activity and related care, and genital hair removal aspects). The variables regarding *genital cleansing and washing* were time away from home, baths per day, frequency of genital hygiene a day, products used in genitalia, posturinary method of hygiene, postevacuation method of hygiene, and vaginal douching. The variables of *sexual activity and related care* were frequency of intercourse per week, habits of having more than one intercourse a day, oral sex, anal sex in the last 30 days, use of lubricant, erogenous substance or sex toys use, vaginal douching after sex, genital cleansing method used before and after intercourse. The variables of *genital hair removal aspects* were frequency, area, and method of genital hair removal and products used before, during, and after hair removal. All variables were comparatively studied both on groups with and without VVC and/or BV.

## Technique

In a gynecological examination, the vaginal pH was determined using a pH indicator paper (colorimetric pH strips; Merck Laboratories, Germany), which was placed for 1 minute on the right side of vaginal wall. Then, vaginal material was collected from the left side of the vaginal wall using two swabs, one designed for smear slide examination under optic microscopy, and a Whiff test, which was considered positive when it released a bad odor after the addition of potassium hydroxide and the other to smear into a Sabouraud agar culture media. The BV diagnosis was performed analyzing the vaginal content smears slides (gram-stained) under oil immersion objective (1,000x magnification) and graded as per the Nugent criteria. 10 Specifically for this study, the diagnosis of BV and VVC were very rigorous, being considered positive only when all three criteria (Nugent score, pH, and Whiff test) were positive. The diagnosis of VVC was positive for women who presented: 1 - symptoms and/or signs of vaginitis, that is, vaginal discharge (described as thin or thick like cottage cheese, with no particular odor), itch or discomfort, external dysuria, and vulvovaginal erythema, 2 - spores, hyphae, or yeast buds identified on microscopic analysis, and 3 - fungal positive culture in Agar Saboureaud medium, read after 36 to 48 hours and until 80 hours of incubation at 25 to 30 degrees Celsius. 11 Women without CVV and/or BV were those who did not fit into the earlier diagnosis and with a vaginal content smear without present inflammation. Women with some symptoms, such as vaginal discharge, sporadic burning or itching, or other clinical symptoms without any laboratory positive tests were included in the control group. Microbiologists performed all laboratory assessments.

## Statistical Analysis

The sample size calculation was based on the prevalence of some genital hygiene habits of women with and without bacterial vaginosis, observed in a previous study. 12 Common variables examined, such as *front to back cleaning of genital area,* presented a 37.3% of prevalence for *never* and 22.9% for *always* among women with BV, and *frequency of vaginal intercourse (less than 7 times per week)* was prevalent on 39% of women as *never* and 22.6% as *always* . Adjusting for the design effect and considering a significance level of 5% and test power of 80%, the sample sizes calculated for the 2 questions were respectively of n = 256 and n = 248. Therefore, the sample should have enrolled at least n = 256 subjects, for there should be half in each group (128 of women with and 128 without VVC and/or BV). The obtained data were analyzed using the SAS version 9.2 SAS Institute Inc, 2002–2008, (SAS Institute, Cary, NC, USA), and the chi-squared 2 and Fisher tests assessed the significance of associations between categorical variables. Demographic and gynecological categorical data are showed as frequency and percentage, and numerical variables are showed by mean ± standard deviation. The categorical variables on genital care and habits were compared between the groups using the chi-squared or Fisher exact test. The significance level was considered *p* < 0.05. The numerical data were compared using the Mann-Whitney test, because of non-normal distribution of data. Both uni and multivariated logistic regressions were used to evaluate the association between the presence of VD and genital hygiene and care habits, and the confidence interval for odds ratio was 95%.

## Results

**Fig. 1.**
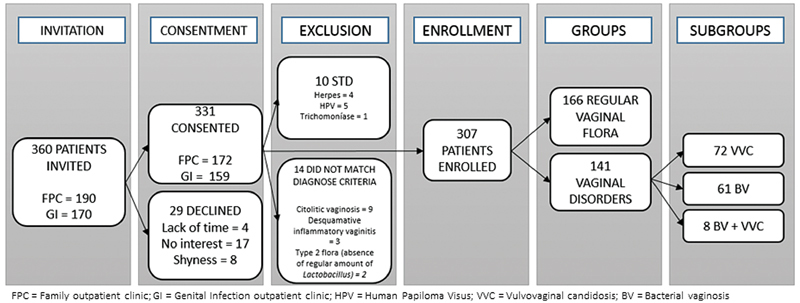
Sampling flow chart.

Twenty-nine out of 360 eligible women (18 from family planning and 11 from genital infections disease outpatient clinics) declined to participate in the study. Of the 331 who volunteered, 10 were excluded from the sample because they presented an associated sexual transmitted disease and 14 that did not fulfill the absence or presence of diagnosis of BV or VVC only, as proposed in this study. Then, 307 participants composed the sample, divided into two major groups: health controls, without any vulvovaginal symptoms (n = 166–54.07%) and women with either VVC and/or BV (total of 141–45.93%), BV (n = 72), VVC (n = 61), and BV + VVC (n = 8) ( Figure 1 ).

The participants were young (33 ± 6.9 years old), with a health weight (body mass index of 22.2 ± 5.5), had 10.2 (± 3.3) years of schooling and one pregnancy. Having no sex partner on last 6 months was reported by 8.5% of women, 83.7% had a steady partner and 7.8% had an eventual partner. Those characteristics did not differ when total of participants were divided according to positive or negative diagnosis for VVC and/or BV. Contraceptive methods, menstrual cycle and smoking habit were also similar between the two groups ( Table 1 ). There were no statistical differences between the studied groups when clinically symptomatic women with negative laboratory results included in control group (regular vaginal flora) were isolated from statistical analysis ( *p* > 0.05).

**Table 1:** Demographic, gynecological and behavioral characteristics of women with and without (controls) VVC and/or bacterial vaginosis

	Total	Controls	VVC/BV	*p* -value *
Variables	(n = 307)	(n = 166)	(n = 141)	
AGE ± SD	33.1 ± 6.9	33.6 ± 6.7	32.6 ± 7.0	0.22
BMI ± SD	22.1 ± 5.5	22.3 ± 5.3	22.8 ± 5.7	0.39
SCHOOLING(YS) ± SD	10.2 ± 3.3	10.4 ± 3.3	10 ± 3.3	0.30
PREGNANCIES ± SD	1.8 ± 1.1	1.8 ± 1.1	1.8 ± 1.2	0.87
SEXUAL PARTNER (SP)				0.91
NO PARTNER	26 (8.5%)	14 (8.4%)	12 (8.5%)	
STEADY PARTNER	257 (83.7%)	140 (84.3%)	117 (83%)	
EVENTUAL PARTNER	24 (7.8%)	12 (7.2%)	12 (8.5%)	
WHITE RACE	162 (52.8%)	87 (52.4%)	75 (53.2%)	0.89
CATHOLIC RELIGION	158 (51.4%)	78 (46.9%)	80 (56.7%)	0.08
CONTRACEPTIVE METHODS 0.31				
NOTHING OR DEFINITIVE	14 (4.6%)	7 (4.2%)	7 (5%)	
BLOCKAGE	21 (6.8%)	13 (7.8%)	8 (5.7%)	
HORMONAL	195 (63.5%)	111 (66.9%)	84 (59.6%)	
IUS - LNG OR CU	77 (25.1%)	35 (21.1%)	42 (29.8%)	
SMOKING	33 (10.7%)	13 (7.8%)	20 (14.2%)	0.07
MENSTRUAL CYCLE +	194 (63.2%)	92 (55.4%)	102 (72.3%)	0.47

Abbreviations: BMI, body mass index; Cu, copper bearing; IUS, intrauterine system; LNG, levonorgestrel; NV, negative vulvovaginitis diagnostic; PV, positive vulvovaginitis diagnostic; SD, standard deviation; VV, vulvovaginitis.

* Chi-square Test, SP for 6 month or more.

There was a significant association between presence of VVC and/or BV and the use of bactericidal soap for daily genital hygiene ( *p* < 0.001, OR = 5.47, IC 95% OR = 2.17–13.8). In contrast, intimate soap (liquid and slightly acid soap) use for daily hygiene and moist wipes use for hygiene after urination were significantly more common among women without VVC and/or BV ( *p* = 0.04, OR = 0.67, IC 95% OR = 0.75–0.98 and *p* = 0.01, OR = 0.24, IC 95% OR = 0.05–0.52, respectively). Mean time away from home, number of baths per day, frequency of genital hygiene, vaginal douching or genital hygiene performed after sexual intercourse did not differ between women with and without VVC and/or BV. Anal hygiene was practiced in the wrong way (from back to front) by 6.6% of the VD-negative group and 11.3% of the VD-positive group, although it was not statistically significant ( Table 2 ). There were no statistically significant differences for these variables when the group of women without vaginal disorders was compared with BV and VVC groups alone.

**Table 2:** Daily habits and products used in genital hygiene by volunteers with and without vulvovaginal disorders

Variables	Total	VD-negative (n = 166)	VD-positive (n = 141)	*p* -value	OR	CI 95% OR
TIME AWAY FROM HOME				0.42*	1.2	0.7–1.82
≤ 5H	109 (35.5)	61 (36.8)	48 (34)			
6H–9H	107 (34.8)	55 (33.1)	52 (36.9)			
≥ 10H	91 (26.6)	50 (30.1)	41 (29.1)			
BODY BATHS PER DAY				0.58*	1.36	0.76–2.92
≤ ONE		36 (21.7)	24 (17)			
TWO		110 (66.3)	100 (70.9)			
≥THREE		20 (12.1)	17 (12.1)			
FREQUENCY OF FG HYGIENE				0.47*	1.49	0.68–3.22
≤ ONE		24 (14.5)	14 (9.9)			
TWO		97 (58.4)	88 (62.4)			
≥THREE		45 (27.1)	39 (27.7)			
FG HYGIENE PRODUCTS						
NOTHING		1 (0.6)	1 (0.7)	1**	—	
BACTERIAL SOAP		6 (3.6)	24 (17)	< 0.0001*	5.47	2.17–13.81
COMMON SOAP		100 (60.2)	80 (56.7)	0.62*	—	
INTIMATE SOAP		59 (35.5)	35 (24.8)	0.04*	0.67	0.75–0.98
BODY LOTION		10 (6)	13 (9.2)	0.89*	—	
OTHERS		6 (3.6)	8 (5.7)	0.38*	—	
POST URINARY HYGIENE						0.05–0.52
USE OF DBP		139 (83.7)	123 (87.2)	0.32	—	
WASHES WITH SOAP		18 (10.8)	14 (9.9)	0.47	—	
USES MOIST WIPES		18 (10.8)	6 (4.3)	0.01	0.24	
OTHER		2 (1.2)	1 (0.7)	0.56	—	
NOTHING		0 (0)	2 (1.4)	–	—	
POST EVACUATION HYGIENE					0.94	0.6–1.7
FB DBP USE		148 (89.2)	119 (84.4)	0.41		
BF DBP USE		11 (6.6)	16 (11.3)	0.52		
WASH WITH WATER		43 (25.9)	37 (26.2)	0.54		
SOAP		21 (12.7)	24 (17)	0.48		
NOTHING		1 (0.6)	2 (1.4)	0.80		
VAGINAL DOUCHING					0.92	0.4–2.11
NEVER		112 (67.5)	96 (68.1)	0.26		
SOMETIMES		14 (8.4)	11 (7.8)	0.54		
ALWAYS		40 (24.1)	34 (24.1)	0.48		
FG HYGIENE BEFORE SI					1.25	0.75–2.07
NO		41 (27.7)	41 (32.5)	1.00		
YES		107 (72.3)	85 (67.5)	0.11		
FG HYGIENE AFTER SI					1.3	0.59–2.86
NO		13 (8.8)	14 (11.1)	0.84		
YES		135 (91.2)	112 (88.9)	0.14		

Abbreviations: Bact., bactericide; BF, back to front; CI, confidence interval; DBP, disposable bathroom paper; FB, front to back; FG, female genital; Min., minute; NV, negative diagnostic of vulvovaginitis; OR , odds ratio confidence interval of 95% for risk of vaginal disorder; PV, positive diagnostic of vulvovaginitis; SI, sexual intercourse; VC, vulvovaginal candidiasis; VC, vaginal disease; VV, vulvovaginitis.

Observation: Inconsistence in numbers might occur due to a number of volunteers who did not have sexual intercourse in the last 6 months and were not included in related questions and women who could use more than hygiene products or way of cleaning themselves.

Chi-squared test* and Fisher** exact test were used for p value.

Anal sex practiced on the 30 days preceding the interview was reported by 30.2% of women in the VD group, and in 8.8% in the group without VD ( *p* < 0.0001, OR = 4.34 IC 95% OR = 2.21-8.55). Comparing the groups of women with BV and/or VVC to those without VD, it was observed that both anal sex ( *p* < 0.001, O.R = 2.33 IC 95% OR = 1.08–5.05) and use of sex toys ( *p* < 0.03, OR = 2.33, IC 95% OR = 1.08–5.05) correlated to the presence of bacterial vaginosis. Among the 51 women reporting anal sex in the last 30 days and the 31 reporting the use of sex toys, only 2 (6.45%) of the first and 2 of the second (4%) groups used condoms regularly. Frequency of sexual intercourse, oral sex practice and lubricant use were statistically similar between groups ( Table 3 ).

**Table 3:** Sexual habits of women with and without vulvovaginal disorders

	VD-negative	VD- positive	*p* -value	OR	CI 95% OR
Variables	(n = 166)/n (%)	(n = 141)/n (%)			
SI PER WEEK,			0.49*	1.55	0.61–3.95
NO SI (#)	18 (10.8)	15 (10.6)			
< ONCE	39 (23.5)	35 (24.8)			
1–3 TIMES	92 (55.4)	69 (48.9)			
≥ 4	17 (10.2)	22 (15.6)			
+ 1 SI/ DAY			0.8*	1.3	0.59–2.93
NEVER	99 (66.9)	81 (64.3)			
SOMETIMES	36 (24.3)	31 (24.6)			
FREQUENTLY	13 (8.8)	14 (11.1)			
ORAL SEX (receptive)			0.9*	1.08	0.52–2.26
NEVER	84 (54.5)	67 (51.9)			
SOMETIMES	52 (33.8)	46 (35.7)			
FREQUENTLY	18 (11.7)	16 (12.4)			
ANAL SEX (IN THE					
PREVIOUS 30 DAYS)			< 0.0001*	4.34	2.21–8.5
NO	135 (91.2)	88 (69.8)			
YES	13 (8.8)	38 (30.2)			
USE OF LUBRICANT			0.8*	1.0	
NO	118 (79.7)	99 (78.6)			
YES	30 (20.3)	27 (21.4)			
EROGENOUS SUBSTANCE OR SEX TOYS USE			0.03*	2.33	1.1–5
NO	137 (92.6)	106 (84.1)			
YES	11 (7.4)	20 (15.9)			
DOUCHING AFTER SI			0.78*	1.0	0.58–1.8
NEVER	101 (60.8)	88 (62.4)			
SOMETIMES	14 (8.4)	9 (6.4)			
FREQUENTLY	51 (30.7)	44 (31.2)			

Abbreviations: BV, bacterial vaginosis; CI, confidence interval; ns, *p* -value not significant; OR, odds ratio; SI, sexual intercourse; VC, vaginal candidiasis; VD, vulvovaginal disorders;.

(*) Chi-squared and (**) Fisher test, ( #) for more than 6 months.

The great majority of the participants (95%) reported to have genital hairs removed, with no significant statistical difference between groups. The characteristics of this habit, such as reason for epilation, method, frequency, area of epilation, and products used before and after having genital hair removed were similar among the different groups ( *p* > 0.05). Although also similar between groups, the opinion of the 61% of the participants about the influence of hair removal to genital health were highly reported as *probably harmful* , and most of those who removed their genital hairs reported an associated vulvar irritation increase because of this practice (71% of women without VD and 78% of women with VD). The results showed that independent of the technique or area of genital hair removal, this practice did not relate to the presence of genital infections.

## Discussion

Our results showed that bactericidal soap, habit of having anal sex, and using sex toys during intercourse were related to a higher prevalence of VVC and/or BV, while other variables, such as genital hair removal, oral sex, use of lubricants, frequency of sexual intercourse, or other hygiene habits did not show such association. In comparison to other studies with similar objectives, 7 9 12 this study investigated many variables for the first time. Probably because the studied population lives in a tropical weather country, this explains why most of women are used to bathing twice per day. However, less than 15% of them had the habit of washing the genitalia other than when bathing, and the common, bar-shaped soap was the most used cleanser to do daily genital hygiene. However, these findings agree to another study 13 investigating the skin care regimen of 121 pregnant volunteers who reported to take more than one bath a day and to use common bar soap to do genital hygiene.

On the other hand, Volochtchuk et al. 14 evaluated the pH of 42 different forms of soap and found that most bar-shaped soaps had a pH between 9 and 10, while liquid soap had a pH lower than 8. Gfatter et al. 15 underlines that an alkaline pH is the main factor to provoke irritation and skin dehydration as well as to eliminate local protection. In fact, genital hygiene with intimate soap (liquid and with slight acidity), the second most commonly used product for this purpose, was more frequently reported by women without VVC and/or BV ( *p* < 0.05). Schmid and Korting 16 suggested that the lower pH helps to maintain the physiological acid coat of the skin, thus preventing the installation of pathogens. Nevertheless, it is important to highlight that when compared with healthy ones, women with VVC and/or BV used bactericidal soap more frequently. The probable explanation is that the sterilization of vaginal flora that this product causes, including its protective microorganisms, which, once eradicated, offer no dispute for nutrients or substrate, leading to rainless growth of harmful bacteria, and, therefore, to the increase in vulvar and vaginal infections.

Some previous studies indicate more frequent habits of vaginal douching among women with vaginal infections. 17 18 We investigated vaginal douching in two situations: as a daily habit and after sexual intercourse. In the first case, 24% of women answered to do it as a habit and 31% reported to do it only after intercourse. Nevertheless, we found the same rates for both with and without VVC and/or BV groups. Other Brazilian studies report rates from 20 to 40% and do not support the association between vaginal douching and genital infections. 19 20 21

Despite our population having been selected from a low-income public hospital, the average of 10 years of schooling might explain the avoidance of back-to-front handling of disposable toilet paper after evacuation in 6.6% of the VD-negative group and in 11.3% in the VD-negative group. This data was similar to the one found by Cesar et al. 20 when investigating pregnant women, who found rates between 9 and 11%. The low prevalence of women performing back-to-front wiping found in this study probably explains the lack of correlation between this variable and the presence of VD. Disposable toilet paper was the prevalent mode of hygiene posturination and postdefecation. Our findings showed much lower rates for washing after toilet use (10–26%) than those found in a study that enrolled American women, who seemed to have the habit to wash their genitalia with water and soap after urination and defecation in 50 to 66% of the time. 22 Interestingly, we found a statistically significant difference for the use of moist wipes after urination pointing it as a more frequent habit of women without genital infections ( *p* < 0.05). This finding agrees with literature, which sustains the use of moist wipe as safe and beneficial to genital health. 23 Because of its moist characteristic, these wipes might be more efficient at promoting genital hygiene than regular disposable toilet paper.

Sexual practice differed between women with and without infections in the modalities anal sex in the previous 30 days to the date of data collection and use of sex toys during intercourse. Both were more practiced by women with BV ( *p* < 0.0001 and *p* = 0.03, respectively). Although there was a relatively high reporting of anal sex, no one reported being paid for sex. This is probably an underestimated prevalence in the literature as anal sex is yet a taboo, and, perhaps, the fact of using a self-reported survey contributed to the increased numbers of such a reporting. In addition, there is a possibility of women with vulvovaginitis having had opted for anal penetration because their vaginas were sore. Thus, this data agrees with the findings of Rosa and Rumel 24 that pointed statistical significance for the relation between anal sex and clinically diagnosed VVC and/or BV. It is known, however, that the anal region is colonized by bacteria that, once transmitted to the genital region, can be quite harmful to its environment. Although our study did not investigate whether those women had the habit of anal sex before or after (or even alternating) having vaginal penetration, gynecologists should educate their patients about changing the condom used to anal sex before having vaginal penetration, or not going from anal to vaginal penetration afterwards. The anal sex data presented in this paper (30.2% in the VD-positive group versus 8.8% in the VD-negative group) is similar to the one from the American women population between 15 and 44 years, reported by Chandra et al. 25 in 2013.

Investigation about the use of sex toys is as scarce in the medical literature, as the physiopathology knowledge of its role over the disturbance of vaginal homeostasis and reliability of its potential disturbance. A study conducted in China with homosexual women found use of sex toys in 13.4% of the time, but half of the time they did not use a condom protecting it. Although our population have declared their selves as heterosexual, the frequency of sex toys use is similar to the one found in this study (11.3%). 26 The literature 8 9 has suggested that oral sex can play an important role in sexually transmitted diseases, and Saini et al. 9 concluded, in their study, that “oral sex is a mode of transmission for genital pathogens”. However, our results showed equal prevalence in oral sex (both giver and receptor) for women with and without VD.

There were no statistically significant differences in terms of habit, frequency, or method of genital hair removal among the studied groups. However, most women in this study declared to associate vulvar irritation with removal of genital hair. Even though it can present no harm to vaginal health, this symptom can often mimic VD symptoms, which might confound the patient or even mislead the diagnosis and treatment. In addition, it is important that women opt for hair removal practices that promote as little discomfort as possible.

The limitation in this study includes the fact that the variables of genital care were self-reported and obtained through a questionnaire not yet validated in the medical literature, which may have led to under reporting and misclassification of some behaviors. However, the inclusion of a great number of women and the accuracy applied in the methodology for vaginal disorders diagnose might have balanced any inconsistency. Further studies containing the patient's correct diagnosis in conjunction with hygiene and genital care habits and associated complaints would be valuable to guide future orientation improving the prevention and treatment of vaginal disturbances.

## Conclusion

The results suggest that some hygiene habits and the absence of others were associated with the presence of VVC and/or BV. It is not clear, though, if female genital hygiene can be the cause or consequence of such VDs. Controlled trials are needed to clarify the influence of hygiene and sexual habits on the vulvovaginal environment.
